# Selenomethionine: A Pink Trojan Redox Horse with Implications in Aging and Various Age-Related Diseases

**DOI:** 10.3390/antiox10060882

**Published:** 2021-05-31

**Authors:** Muhammad Jawad Nasim, Mhd Mouayad Zuraik, Ahmad Yaman Abdin, Yannick Ney, Claus Jacob

**Affiliations:** 1 Division of Bioorganic Chemistry, School of Pharmacy, Saarland University, D-66123 Saarbruecken, Germany; jawad.nasim@uni-saarland.de (M.J.N.); s8mhzura@stud.uni-saarland.de (M.M.Z.); yaman.abdin@uni-saarland.de (A.Y.A.); yannick.ney@uni-saarland.de (Y.N.); 2 University Lille, CNRS, Centrale Lille, University Artois, UMR 8181–UCCS–Unité de Catalyse et Chimie du Solide, F-59000 Lille, France

**Keywords:** aging, selenium, selenomethionine (SeMet), reactive selenium species (RSeS)

## Abstract

Selenium is an essential trace element. Although this chalcogen forms a wide variety of compounds, there are surprisingly few small-molecule organic selenium compounds (OSeCs) in biology. Besides its more prominent relative selenocysteine (SeCys), the amino acid selenomethionine (SeMet) is one example. SeMet is synthesized in plants and some fungi and, via nutrition, finds its way into mammalian cells. In contrast to its sulfur analog methionine (Met), SeMet is extraordinarily redox active under physiological conditions and via its catalytic selenide (RSeR’)/selenoxide (RSe(O)R’) couple provides protection against reactive oxygen species (ROS) and other possibly harmful oxidants. In contrast to SeCys, which is incorporated via an eloquent ribosomal mechanism, SeMet can enter such biomolecules by simply replacing proteinogenic Met. Interestingly, eukaryotes, such as yeast and mammals, also metabolize SeMet to a small family of reactive selenium species (RSeS). Together, SeMet, proteins containing SeMet and metabolites of SeMet form a powerful triad of redox-active metabolites with a plethora of biological implications. In any case, SeMet and its family of natural RSeS provide plenty of opportunities for studies in the fields of nutrition, aging, health and redox biology.

## 1. Introduction

Selenium is an essential trace element in humans [1]. Since its discovery by Joens Jacob Berzelius (1779–1848) in 1817, the element has fascinated chemists and biochemists alike. During the last five decades, hundreds of organic selenium compounds (OSeCs) have been produced to mimic the unique biological activity of selenium enzymes, such as the human glutathione peroxideases (GPx), human thioredoxin reductase (TR) and iodothyronine deiodinase (ID) [2,3]. Notably, nature itself produces only a few organic selenium compounds on its own. Besides the rather prominent amino acid SeCys and intermediates involved in its biosynthesis from inorganic selenite SeO_3_^2−^ and subsequent metabolic degradation to methylselenide CH_3_SeH, dimethylselenide (CH_3_)_2_Se and dimethylselenonium (CH_3_)_3_Se^+^, such natural small-molecule selenides are rare. The few notable exceptions found Nature include compounds such as the histidine derivative and ergothionine analog selenoneine present in tuna fish and inorganic selenocyanate (SeCN^−^) in the green freshwater algae *Chlorella vulgaris* [4,5].

**Figure 1:**
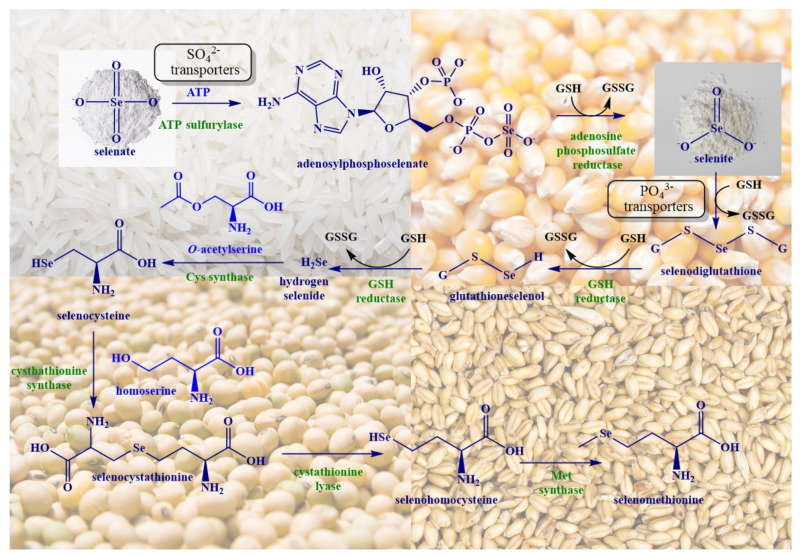
Biosynthesis of SeMet in plants involves the uptake of SeO_4_^2−^ via SO_4_^2−^ transporters and its interaction with ATP to produce adenosylphosphoselenate, which is reduced to SeO_3_^2−^ by adenosylphosphosulfate reductase. SeO_3_^2−^, either absorbed via PO_4_^3−^ transporters or reduced from SeO_4_^2−^, is further reduced to selenide by GSH via intermediates such as selenodiglutathione and glutathioneselenol. The interaction of selenide with *O*-acetylserine results ultimately in the formation of SeCys. Interaction of SeCys with homoserine in the presence of cystathionine synthase generates selenocystathionine which is cleaved enzymatically by cystathionine lyase to selenohomocysteine, pyruvate and ammonia. This is followed by the formation of SeMet from selenohomocysteine via methionine synthase.

**Figure 2:**
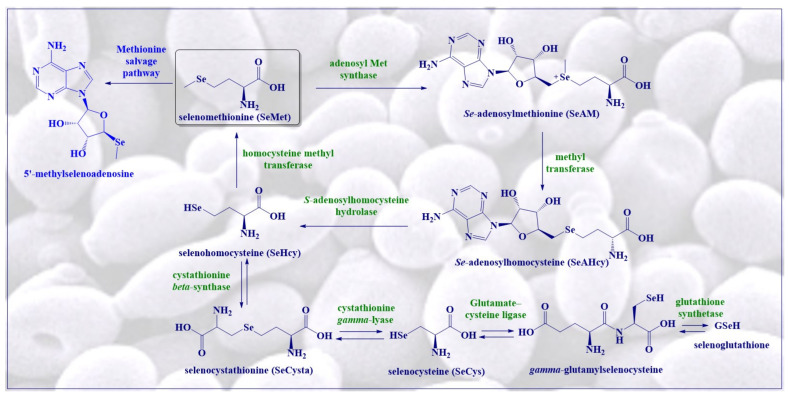
Structures of some of the metabolites of SeMet found in selenium-enriched yeast.

Indeed, SeCys dominates most discussions of selenium in proteins and enzymes, which is hardly surprising, as most of the roughly 25 selenium proteins identified in humans to date contain one or more SeCys residues [6]. In fact, SeCys is often referred to as the 21st amino acid, and its insertion into human proteins is controlled tightly via specific SeCys insertion sequences (SECISs). In contrast, the other natural selenium amino acid, selenomethionine (SeMet), occupies a more exotic niche in biology, although SeMet combines some truly amazing and exciting features worth discussing. SeMet, for instance, is produced in plants and fungi and in many organisms literally sneaks into proteins and enzymes in place of its sulfur analog methionine (Met), thereby endowing these proteins with extra redox activity [7]. SeMet on its own is also highly redox active, similar to the flagship antioxidant ascorbic acid, and in yeast is a fine source of numerous, equally active metabolites, such as selenohomocysteine, selenoglutathione, *γ*-glutamylselenocysteine and *Se*-adenosylselenohomocysteine. In humans, SeMet is absorbed via intestinal transport channels and subsequently enters the methionine pool, where it is stored and consequently recruited from to become integrated into proteins [8]. Once in the liver, SeMet follows the methionine cycle and *trans*-selenation pathways to produce *S*-adenosyl SeMet, adenosyl selenohomocysteine, homocysteine, selenocystathionine and SeCys, as shown in Figure 1 and Figure 2 [9].

As part of this minireview, we shall therefore provide some essential information about this fascinating and in many aspects unique proteinogenic amino acid, from its biosynthesis in plants and metabolism in yeast to its ability to replace Met in proteins and its role as a food supplement. From the onset, we would like to emphasize that we have focused on some of the more recent and exciting discoveries in this field and therefore shall refer to the existing literature for more basic information [10,11,12,13].

## 2. Biosynthesis of SeMet in Plants

Higher organisms, such as mammals and humans, are unable to synthesize SeMet. This task is left to plants and fungi, including yeast (*Saccharomyces cerevisiae*) and a few edible mushrooms, such as the Shiitake (*Lentinula edodes*) and King Bolete (*Boletus edulis*) mushrooms [14,15]. Plants absorb SeO_3_^2−^ and SeO_4_^2−^ from the soil and convert these inorganic salts to different organic forms of selenium following a sequence of biochemical events, as shown in Figure 1. The absorption of SeO_4_^2−^ and SeO_3_^2−^ in plants is controlled tightly and involves certain transporters, such as sulfate (SO_4_^2−^) and phosphate (PO_4_^3−^) transporters in rice and wheat, respectively [16,17,18]. SeO_4_^2−^ interacts with ATP in the presence of ATP sulfurylase (ATPS) to produce adenosylphosphoselenate, which is subsequently reduced by adenosine phosphosulfate reductase to SeO_3_^2−^ consuming the reduced form of glutathione (GSH) as the electron donor [19]. SeO_3_^2−^, either absorbed via PO_4_^3−^ transporters or reduced from SeO_4_^2−^, interacts spontaneously with GSH to produce selenodiglutathione (GSSeSG), which is subsequently reduced by GSH reductase (GSR) to generate glutathioneselenol (GSeH), which is again reduced by GSR in the presence of GSH to form hydrogen selenide (H_2_Se) [20]. The interaction of H_2_Se with *O*-acetyl serine in the presence of cysteine synthase results in the formation of SeCys [21].

SeMet is synthesized from SeCys in several steps. The α-amino acid homoserine interacts with SeCys to produce a selenocystathionine adduct, which is subsequently hydrolyzed to selenohomocysteine, pyruvate and ammonia, as illustrated in Figure 1. Selenohomocysteine is then converted to SeMet by methionine synthase [22]. There are two types of methionine synthase, i.e., cobalamine-dependent methionine synthase (E.C. 2.1.1.13) and cobalamine-independent methionine synthase (E.C. 2.1.1.14). The main function of both methionine synthases involves the catalysis of the transfer of functional methyl groups from 5-methyltetrahydrofolate to the thiol moiety of (seleno)homocysteine to produce tetrahydrofolate and (Se)Met [23]. Among the various plant species, cereals and forage crops primarily convert Se to SeMet, which they subsequently store. These plants also incorporate SeMet into proteins in lieu of Met, as the tRNA^Met^ responsible for the incorporation of Met does not distinguish between Met and SeMet, an issue of the blatant hijacking of a sulfur pathway by selenium we shall discuss on several occasions during this review. Indeed, the production of SeMet in plants is not linked to any specific stimuli or requirements and is mainly running side by side to the synthesis of Met [24]. Whether Met or SeMet are synthesized then depends mostly on the amount of selenium available in the soil and not on any sophisticated design or game plan(t).

Yeast as a eukaryote can reduce SeO_3_^2−^ and synthesize SeMet following the footsteps of plants, although it also produces other selenium species, including elemental red selenium Se^±0^, often in form of nanoparticles, a metabolic detoxification pathway actually also common in many bacteria and fungi, including *Lactobacillus plantarum, Escherichia coli* and *S. cerevisiae* [25]. Compared to plants and yeasts, mammals are unable to synthesize SeMet, as they turn SeO_3_^2−^ into GSSeSG, H_2_Se and SeCys, yet do not process SeCys to SeMet [9].

## 3. SeMet in Yeast

The nonspecific and accidental biosynthesis of SeMet in yeast from SeO_4_^2−^ or SeO_3_^2−^ follows the one in plants [26]. Interestingly, yeast is also able to take up SeMet quite readily from external sources. Once inside the yeast cell, SeMet plays several rather special roles. As some metabolic enzymes are unable to distinguish between Met and SeMet, just as in plants, this enables SeMet to take on the false identity of its sulfur analog and literally to sneak into proteins in place of Met [27,28]. It is important to mention that the amount of total Met in yeast (*S. cerevisiae)* is very low, i.e., around 0.75%, based on dry weight [29]. The extent to which Met is replaced by SeMet depends upon the conditions in which yeast is cultivated. Under standard industrial conditions, 30–45% of Met can be substituted by SeMet, while controlled laboratory-based experimental conditions may yield an amazing >98% substitution of SeMet for Met in the entire protein pool, as found, for example, in a wild-type yeast [30,31].

Furthermore, in such fungi, SeMet can requisite the machinery responsible for changing Met into many other natural sulfur compounds, and this results in the production of a plethora of interesting selenium compounds, some of which are shown in Figure 2.

Liquid chromatography coupled with orbitrap mass spectrometry, for example, has been utilized to hunt for such OSeCs in *S. cerevisiae* treated with SeO_3_^2−^, SeO_4_^2−^ and SeMet [32]. The highest amount of Se inside cells has been observed in yeast cells treated with SeMet as compared to SeO_3_^2−^ or SeO_4_^2−^. Treatment of yeast with SeMet significantly enhances the intracellular level of SeMet in yeast in less than 15 min, confirming a surprisingly fast and comprehensive uptake of SeMet by these cells. SeO_3_^2−^ and SeO_4_^2−^ are also taken up and processed in yeast to organic selenides, such as selenohomocysteine (SeHcy), *gamma*-glutamylselenocysteine, selenoglutathione (GSeH), 5′-methylselenoadenosine, *Se*-adenosylhomocysteine (SeAHcy) and *Se*-adenosylmethionine (SeAM), in addition to elemental selenium as mentioned before.

Notably, while yeast can synthesize SeMet following the sulfur pathway, it is not the preferred selenium compound produced in *S. cerevisiae*. Popular notions that selenium-enriched yeast contains mostly SeMet therefore are not entirely correct. Among the various selenium compounds found in this fungus, the rather exotic SeAHcy shown in Figure 2 dominates the field, as it amounts to around 70% of total selenium content [32]. In contrast, the individual shares of other selenium compounds are below 10%. Interestingly, SeAM is also formed in yeast and can take on some of the roles of its more popular sulfur analog, *S*-adenosylmethionine (SAM), which acts as a methyl donor in various eukaryotic cells. SeAM serves, for example, as a precursor in the synthesis of SeAHcy and is also involved in the methylation of lipids, proteins, nucleic acids and various secondary metabolites. Furthermore, methyltransferases are able to transfer methyl groups from SeAM to different nucleic acids, i.e., ribosomal and transfer RNAs and even DNA [33].

SeAHcy is hydrolyzed by *S*-adenosylhomocysteine hydrolase to adenosine and SeHcy, as shown in Figure 2. Unlike methionyl-tRNA synthetase, which does not discriminate between Met and SeMet, *S*-adenosylhomocysteine hydrolase actually demonstrates a slight discrimination, as its hydrolase activity against SeAHcy is relatively low as compared to its activity against *S*-adenosylhomocysteine. This also explains why SeHcy is found in yeast in relatively small amounts of around 8.5% of selenium compounds, despite the presence of excessive amounts of SeAHcy, for instance in yeast cells treated with SeMet. The presence of 5′-methylselenoadenosine, a rather exceptional selenide shown in Figure 2, in such cells treated with SeMet has been reported [34] Considering the overall situation in yeast, SeMet follows the same metabolic pathways as Met, although the distribution of selenium species differs significantly from that of sulfur analogs. In any case, neither SeMet nor SeHcy is the main selenium compound in *S. cerevisiae*, with more exotic substances such as SeAM and chiefly SeAHcy dominating the field.

## 4. Redox Activity and Catalysis of SeMet

As mentioned already, many organisms are unable to distinguish between SeMet and Met and therefore tend to accept SeMet in many physiological processes in place of Met. This has notable consequences, as SeMet tends to be more reactive than Met and often acts as a redox cycler and catalyst. This strong antioxidant potential associated with selenium is found for SeMet as a free amino acid, for SeMet in proteins and enzymes and, notably, also for a range of other SeMet precursors and follow-on products containing the same selenide RSeR’ motif [35]. Then again, one may mention that the selenide RSeR’ generally is less reactive compared to the selenol(ate) RSeH or RSe^−^. Nonetheless, the literature is rich in examples underlining the biological redox activity of SeMet.

In simple assays, SeMet protects dihydrorhodamine 123 and supercoiled plasmid DNA from oxidation mediated by peroxynitrous acid (ONOOH) [36,37]. This protective effect is attributed to radical-mediated oxidation of SeMet to its oxidized selenoxide form SeMetO [38]. The reaction of SeMet with ONOOH follows a second-order rate constant of *k* ~2.4 × 10^3^ M^−1^s^−1^, which is almost tenfold higher than the one for Met at just 3.64 × 10^2^ M^−1^s^−1^. Not surprisingly, SeMet therefore competes significantly with cellular targets at similar concentrations [38,39]. The oxidation of SeMet to SeMetO is not limited to ONOOH, and an analogous reactivity has also been observed for other oxidants, such as H_2_O_2_, the enzymatic monooxygenase system, amino acid-, peptide- and protein-bound hydroperoxides, hypothiocyanous acid HOSCN and hypochlorite HOCl-derived chloramines [40,41,42]. Moreover, a preference of hydroperoxides (ROOH) for SeMet over Met and GSH has also been noticed, and these hydroperoxides produced in amino acids, peptides and proteins tend to oxidize SeMet quite rapidly, as compared to the standard oxidant H_2_O_2_ [43]. SeMet is therefore a good, albeit not outstanding, antioxidant and may protect proteins against oxidative stress (OS) caused by a range of reactive oxygen species (ROS).

Equally notably, SeMet itself is not only a small-molecule antioxidant, and its ability to become part of proteins and enzymes in the position of Met enables SeMet to be present when and where such oxidative damage may occur. In sharp contrast, the oxygen analog of SeMet and Met, the amino acid methoxinine, is unable to hijack pathways designated for Met, possibly because the oxygen atom is smaller than sulfur and selenium, albeit smaller atoms usually are less demanding and more flexible in such situations. Regardless of the deeper philosophical questions of why Se, and not O, may disguise itself as sulfur, the implications of this Trojan Horse issue include the more or less random replacement of Met by SeMet in proteins, such as human membrane protein B7-1. The glycoprotein B7-1 comprises six Met residues out of a total of 201 amino acids and SeMet has been reported to replace 60% of these Met residues. In another human protein, angiopoietin-2 (Ang2), which contains four Met residues out of a total of 215 amino acids, SeMet has been reported to replace 93% of Met. A similar trend has been reported for other human proteins, including Tie2, netrin-1, plexinB3, ADAM10 and EphA3, where SeMet replaces more than 85% of the Met residues [44,45,46].

**Figure 3:**
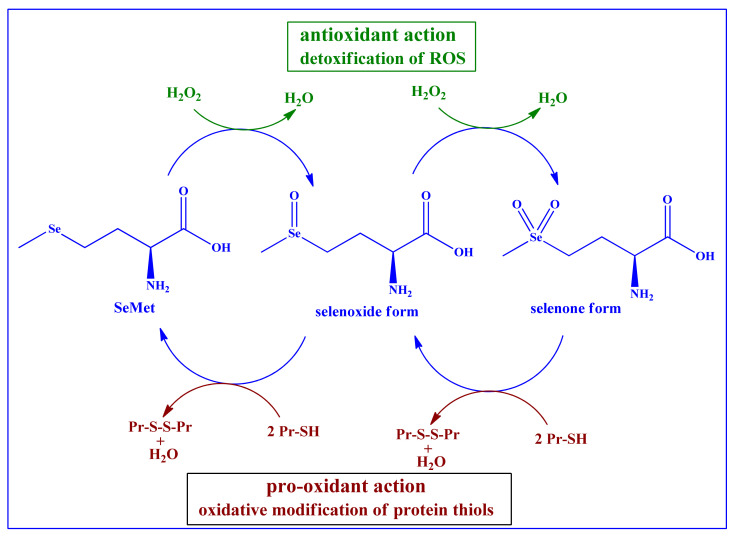
SeMet in its reduced and oxidized forms is redox active and able to engage in catalytic detoxification of ROS, such as H_2_O_2_. The resulting redox cycle consumes thiols and hence may change the cellular concentration of GSH and modulate the cellular thiolstat. Please also note that neither sulfur in Met nor selenium in SeMet are overoxidized under physiological conditions. Therefore, higher and quite interesting oxidation states, such as sulfones (RS(O)_2_R’) and selenones RSe(O)_2_R’) are less common and more an object for ex vivo hypothetical studies (Pr = protein) [47].

This replacement does not go unnoticed, as the biological behavior of SeMet differs from the one of Met. Although both chalcogens are redox active and may be oxidized to sulfoxide (RS(O)R’) and selenoxide (RSe(O)R’), respectively, selenides and selenoxides are usually more (re-)active. The selenide, for instance, is the far better reducing agent and antioxidant compared to the sulfide, hence providing stronger antioxidant protection for the protein containing SeMet in place of Met and also a direct protection for the environment this protein is placed in, as shown in Figure 3. Interestingly, the oxidized form, the selenoxide, is also more reactive compared to the sulfoxide and may react spontaneously yet also selectively with thiol groups, such as the one in GSH. This enables the selenoxide to redox (re-)cycle the selenide whilst producing GSSG and H_2_O, as depicted in Figure 3. The ability of SeMet and thus also SeMet containing proteins to act as redox catalysts has been corroborated recently by an in vitro study conducted by Caroll et al., in which the oxidized form SeMetO has been reduced spontaneously by GSH to recycle SeMet, a process subsequently coupled to energy metabolism via the reduction of oxidized glutathione disulfide GSSG by glutathione reductase in the presence of NADPH with a second-order rate constant *k_2_* ranging from 10^3^ to 10^4^ M^−1^s^−1^. Apart from a spontaneous reduction of SeMetO in the presence of GSH, it also looks as if thioredoxin reductase (TR) has the ability to accept SeMetO as substrate, reducing SeMetO to SeMet [47].

It is therefore not surprising that proteins and enzymes loaded with SeMet exhibit somewhat different and de facto additional properties compared to their unsubstituted analogs. In general, these proteins and enzymes are now also good and often catalytic antioxidants, albeit not as strong as enzymes based on SeCys, such as GPx or human TR.

Taken together, the selenide/selenoxide redox pair therefore is a spontaneous redox cycler and catalyst, while the sulfoxide itself is more or less inert and requires the assistance of enzymes such as sulfoxide reductases MsrA or MsrB to get reduced to sulfide [48].

Please note that not every SeMet found in a plant, fungal or mammalian cell is incorporated into proteins. In humans, dietary SeMet intake corresponds to the total Se content in the blood. Here, SeMet is incorporated primarily by erythrocytes into hemoglobin. In plasma, SeMet is found mostly in the albumin fraction. In humans, SeMet is often associated with albumin. For example, SeMet concentrations of 28.3 and 53.4 ng/g have been reported in individuals residing in a selenium-deficient region of China. This concentration of SeMet corresponds to around 20% of the total selenium content in albumin. In contrast, individuals residing in selenium-rich areas of China exhibited elevated levels of SeMet, which constituted around 47.5% of the total selenium content in albumin [49].

## 5. Implications of SeMet in Aging and Diseases Related to OS

The high redox activity of SeMet has major physiological consequences, which can be classified as the result of (a) SeMet as an antioxidant, (b) SeMet as a GPx-like redox catalyst, (c) SeMet as a provider of selenium, (d) SeMet as an intermediate for a family of RSeS and (e) SeMet as a substitute for Met in proteins and enzymes. In most instances, a medley of these different aspects unique to this selenium amino acid is responsible for the physiological activities recorded in practice. Here, the scope of biological activities of SeMet is extremely broad, including simple selenium supplementation as a nutraceutical, antioxidant, anti-inflammatory and preventive activities. As there are numerous reports on such possible activities in the literature a few selected and rather interesting highlights of such activities need to suffice as a part of this review, with relevant literature provided for further information.

### 5.1. Selenomethionine Supplements Minimize Cardiac Dysfunction

The population above the age of 65 is more prone to suffer from cardiovascular diseases which are occasionally associated with elevated levels of ROS [50]. The main sources of intracellular ROS involve mitochondria which are found especially abundantly in cardiac tissues. The dysregulation of mitochondrial functions and excessive ROS generation may lead to pathologies, such as myocardial ischemia (MI) and myocardial reperfusion (MR) [51]. In these cells, SeMet has been reported to act as an antioxidant able to scavenge these harmful species and therefore to serve as an efficient cardio-protective agent [52]. In a regime of an open-label randomized clinical trial, 100 peripartum cardiomyopathy (PPCM) patients with reduced left ventricular ejection fraction below 45% and reduced total selenium levels below 70 μg/L were provided either with an oral supplement of 200 μg/day of SeMet for three months or a placebo, together with the recommended therapy. The symptoms of heart failure were observed in 69% of the placebo group of 54 patients and in only 39% of the group of 46 patients supplemented by SeMet. A total of 12 deaths from any cause were witnessed during this study, and among them, nine belonged to the placebo group and only three to the supplemented group. Overall, SeMet supplementation in this study therefore decreased the symptoms of heart failure and general mortality [53].

### 5.2. SeMet Regulates Inflammatory Reactions in Atherosclerosis

Aging also interferes with atherogenesis via numerous pathways, and it is highly unlikely that one specific factor dominates the overall pathophysiological mechanism. [54]. Aging is often accompanied by gradual modifications in the bone marrow, which not only leads to enhanced clonal hematopoiesis of indeterminate potential (CHIP), it also provokes alterations of myeloid cell differentiation, and both of these notorious processes trigger atherosclerosis. The progression of CHIP-associated atherosclerosis is mediated by the signaling of IL-6 and activation of the inflammasome. Moreover, aging also leads to a gradual reduction of mitochondrial function and elevated levels of interleukin-6 (IL-6) in the vasculature leading to atherosclerosis. Other factors triggering age-related atherosclerosis include the vasculature and myeloid cells of the immune system which activate the inflammatory pathways, especially IL-6 [54]. Literature demonstrates that selenium is able to downregulate the levels of IL-6 [55,56]. Selenium supplementation has been reported to prevent atherosclerosis by inhibiting OS, modulating inflammation, suppressing endothelial dysfunction and protecting vascular cells against apoptosis and calcification [57]. Furthermore, SeMet stimulates the immune response by reinforcing the endogenous antioxidant defense systems comprising thioredoxin and GSH and by directly scavenging harmful oxidant species. SeMet has also been reported to reduce the formation of atherosclerotic plaques, provide a stable lesion phenotype and enhance the function of vessels [58]. Furthermore, supplementation with SeMet significantly s reduces the accumulation of inflammatory macrophages in lesions [58]. SeMet is also able to modify the acute inflammatory response within a clinically relevant setting [58]. Indeed, these latest developments signify the potentially favorable impact of SeMet supplementation as a therapeutic nutraceutical against atherosclerosis.

### 5.3. SeMet and Viral Infections

Dietary selenium supplementation has been reported to significantly enhance the host immunity against viruses [59]. The topic of a beneficial role of selenium against viral infections has recently gained considerable attention due to the outbreak of COVID-19. It has been reported that individuals residing in selenium-rich areas of China exhibit a better rate of recovery from SARS-CoV-2 infections as compared to individuals residing in selenium-deficient areas in the same country [60]. The deficiency of selenium is generally associated with elevated OS due to decreased detoxification of ROS resulting in hyperinflammation in critically ill patients of COVID-19. Viral infections in general and SARS-CoV-2 infections in particular are also characterized by elevated OS, which ultimately results in immunopathological diseases. Selenium, being a multifunctional trace element, enhances immunity, decreases OS, inhibits viral infections and provides efficient support in healing the patients suffering from COVID-19 [61]. Furthermore, SeMet may inhibit single-stranded RNA viruses, such as Coxsackie virus, hepatitis C virus (HCV) and human immunodeficiency virus (HIV), at a concentration of 50 µM, as observed in in vitro studies [62]. Moreover, SeMet has been reported to inhibit porcine circovirus type 2 (PCV2) in vitro in a concentration-dependent fashion between 2 and 16 µM. PCV2 is a notorious single-stranded DNA virus associated with several complications, including porcine dermatitis nephropathy syndrome (PDNS), postweaning multisystemic wasting syndrome (PMWS), porcine respiratory disease complex (PRDC), congenital tremor type A2 (CT) and pregnant sow reproductive obstruction [63,64,65]. The large differences in concentrations of SeMet required for antiviral activity in these studies against single-stranded RNA and DNA viruses may denote some selectivity of this RSeS towards the later type of viruses, albeit more studies are required to confirm this trend. Overall, SeMet may serve as a multifunctional agent in the fight against viral infections by attacking components of the virus, by serving as an antioxidant and redox modulator, by interfering with the cellular thiolstat and by bolstering the immune system of the host.

### 5.4. SeMet and Cancer

Cancer is not only related to OS, it is also to some extent linked to aging [66]. The prevalence of most cancers increases remarkably with age and cancer is the dominant cause of death in both males and females above 60 years of age [67]. A study in 2018 has reported a median age of 65 for the diagnosis of cancer and a median age of 74 for death due to cancer [67,68]. This consistently increasing risk of developing a certain type of cancer with age has been attributed to the gradual decrease in the microenvironment of many tissues, which is essential for normal tissue maintenance and detoxification, a gradual shift of the balance between healthy and unhealthy cells towards unhealthy cells and a diminished ability of the body to remove dysfunctional cells [66].

Because SeMet is able to counteract each of these changes, it may be beneficial in the fight against cancer in several ways and via several avenues. As discussed already, SeMet is a good antioxidant and may therefore prevent DNA damage. SeMet and its follow-on products are also able to kill cancer cells. Indeed, SeMet is cleaved by the γ—lyase methioninase and therefore produces CH_3_SeH, which in turn causes the oxidation of thiols and generation of superoxide radical anions (O_2_^●−^). Together, these pro-oxidant actions affect the cellular thiolstat and, especially under the conditions of sufficient OS, may also cause apoptosis.

Not surprisingly, SeMet has been reported to inhibit different cancer cell lines, including colorectal (SW-480), prostate (DU-145), breast (MCF-7/S) and melanoma (UACC-375) cell lines [69,70]. In mice, SeMet also inhibits metastasis of breast cancer, while traditional SeO_3_^2−^ supplementation in these studies has triggered extensive metastasis [71].

Notably, a variety of cancer cells depend on a sufficient supply of the amino acid Met, a feature often referred to as the Hoffmann effect [72]. As SeMet is able to substitute for Met, supplementation with SeMet may indeed suppress the uptake and action of Met, thereby replacing the under these conditions less beneficial amino acid Met for the anticancer actions associated with SeMet. If and how such a replacement of Met for the Trojan redox Horse SeMet may be implemented in practice is speculative although worth following with relevant studies.

## 6. Conclusions

Our brief and necessarily incomplete overview of the biochemistry and possible preventive and therapeutic roles of the second selenium amino acid SeMet has shown that this amino acid is as colorful as its more popular relative SeCys. SeMet is produced in plants and certain mushrooms and may also be taken up and processed in mammals and humans. SeMet is not only an excellent supplier of the trace element selenium; it is also redox active and may act as redox catalyst inside cells. Notably, SeMet is very similar to its sulfur analog Met and often uses the cellular pathways of Met, a red herring to allow SeMet to enter a range of sulfur proteins. Evidence for the health benefits of SeMet, for instance, as a selenium-containing food supplement, is therefore mounting, and there are early reports on possible roles in age-related human diseases, such as cardiovascular diseases and atherosclerosis and also during viral infections. The roles of SeMet as the 22nd proteogenic human amino acid are therefore definitely worth more research.
